# Early-Life Biomarkers in Food Allergy Development: A Systematic Review of Immunological and Intestinal Pathways

**DOI:** 10.3390/biomedicines14081661

**Published:** 2026-07-24

**Authors:** Alina-Maria Ivaşko, Corina Ureche, Oana Cristina Marginean, Monica Grama, Teodora Nicola-Varo

**Affiliations:** 1 George Emil Palade University of Medicine, Pharmacy, Science, and Technology of Targu Mures, Gheorghe Marinescu Street 38, 540142 Targu Mures, Mures, Romania; ivasko.alina-maria.25@stud.umfst.ro (A.-M.I.); oana.marginean@umfst.ro (O.C.M.); grama.monica.25@stud.umfst.ro (M.G.); teodora.nicola-varo@umfst.ro (T.N.-V.); 2 Department of Internal Medicine, Emergency Clinical County Hospital of Targu Mures, Gheorghe Marinescu Street 50, 540136 Targu Mures, Mures, Romania

**Keywords:** food allergy, cord blood IgE, fecal calprotectin, fecal zonulin, secretory IgA, predictive biomarkers

## Abstract

**Background:** The increasing incidence of pediatric food allergy (FA) drives an imperative for mechanistically driven risk stratification beyond descriptive diagnostics. The current systematic review aims to assess the predictive and diagnostic value of early-life non-invasive biomarkers: cord blood IgE (CS-IgE), fecal calprotectin (FC), fecal zonulin (FZ) and secretory IgA (SIgA). **Methods:** In accordance with the PRISMA guidelines, we performed a systematic literature search on PubMed/MEDLINE and Scopus for English observational studies published in 2009–2025. The titles and abstracts were screened by two independent reviewers and methodological quality was assessed using the Newcastle–Ottawa Scale (NOS). **Results:** A total of 17 studies met the inclusion criteria. This synthesis demonstrates that CS-IgE reflects a systemic atopic predisposition rather than serving as a highly sensitive predictor for isolated FA. Fecal calprotectin was the most consistent marker of inflammatory activity in non-IgE-mediated food allergies, with median values in food allergy ranging from approximately 200 to 410 µg/g and proven useful in dietary monitoring. Fecal zonulin reflects epithelial barrier dysfunction; however, its clinical utility is limited by suboptimal assay specificity. SIgA primarily reflects mucosal immune maturation and tolerance development rather than serving as a direct diagnostic marker. **Conclusions:** Although CS-IgE, FC, FZ and SIgA exhibit potential in the risk stratification of early FA, their clinical implementation is complicated by methodological heterogeneity and concerns regarding analytical specificity, especially with respect to zonulin assays. We propose an integrated multi-biomarker risk stratification model as a conceptual hypothesis requiring prospective validation in future large-scale studies.

## 1. Introduction

Over the past 20 years, the prevalence of food allergies has increased substantially, representing a growing global public health burden. The rising prevalence has been linked to environmental and lifestyle changes, with genetic predisposition alone being insufficient to explain this trend [1]. Allergies to eggs and cow’s milk protein are common signs of an aberrant immune response to food antigens in newborns and young children, and they have a significant effect on the child’s nutritional development and the family’s quality of life [2,3]. Food allergy is distinct from food intolerance in that intolerance does not develop from immune system dysregulation; for example, lactose intolerance occurs from non-immune factors, such as lactose malabsorption and lactase deficiency [4].

Many environmental and genetic factors contribute to the complex etiology of food allergies. The pathophysiology primarily involves a failure in the establishment of oral tolerance, resulting in an inappropriate immune response to otherwise harmless dietary antigens [5]. This process occurs in two phases: sensitization and elicitation. Beyond the classical Th2 pathway, recent evidence highlights the role of epithelial barrier integrity and epigenetic regulation in food allergy pathogenesis [6,7]. In the sensitization stage, food proteins are captured by antigen-presenting cells (such as dendritic cells) in the gut, skin or respiratory tract, which then results in activation of T-helper 2 (Th2) cells and T-follicular helper cells. These cells facilitate B-cell class switching to produce food-specific IgE antibodies that bind high-affinity IgE receptors (FcεRI) on mast cells and basophils [8,9,10]. However, a considerable percentage of food allergies are not IgE-mediated, which poses a unique diagnostic and prognostic problem [11].

Implementing preventative and early intervention measures requires early prediction of the possibility of developing food allergies. It would be possible to customize proactive strategies and optimize nutritional protocols if high-risk infants were identified prior to clinical signs [12,13].

Food allergies are currently diagnosed using either double-blind placebo-controlled food challenges (DBPCFC), which are expensive and have clinical hazards, or clinical history, skin testing, and serum-specific IgE determination [14]. Emerging biomarker-based approaches, including omics technologies, are being explored to improve diagnostic accuracy and risk stratification [15]. The need for non-invasive biomarkers with good predictive value has led to several investigations into various immunological and inflammatory parameters.

Four classes of biomarkers have attracted the attention of researchers as potential indicators of food allergy risk:

Umbilical cord immunoglobulin E (CS-IgE): The presence of IgE in umbilical cord blood may reflect early atopic susceptibility and intrauterine sensitization of the fetus [16]. Long-term cohort studies have shown that increased levels of CS-IgE are associated with allergy symptoms during childhood and adolescence, showing some sensitivity and specificity depending on the level chosen for diagnosis [17].

Fecal calprotectin (FC): In response to mucosal inflammation, calprotectin, an abundant neutrophil granule protein, is released into the intestinal lumen. High levels of FC have been observed ine children with IgE-mediated and non-IgE-mediated cow’s milk protein allergies [18,19,20]. These findings suggest that epithelial barrier disruption is present in children with allergies and appears to correlate with intestinal mucosal inflammation.

Fecal zonulin (FZ): Zonulin regulates gut permeability and is the single physiological regulator of gut tight junctions [21]. In chronic inflammatory conditions, both locally and systemically elevated zonulin levels have been correlated with decreased mucosal immunological tolerance and increased permeability [22]. Fecal zonulin in children with dysfunctional food tolerance may be a useful non-invasive marker of intestinal barrier integrity.

Secretory IgA (SIgA): Secretory IgA is produced locally in the gastrointestinal tract and is critical for maintaining mucosal tolerance and protecting against pathogenic antigens [23,24]. SIgA levels have been associated with a reduced risk of allergen sensitization, suggesting a protective role in controlling the uncontrolled uptake of dietary antigens early in life [25]. Low concentrations of salivary and breast milk SIgA are associated with increased susceptibility to food sensitization and the development of cow’s milk protein allergy. In contrast, elevated SIgA levels observed during gut dysbiosis may indicate compensatory mucosal immune activation rather than genuine immunological protection [26,27,28].

These four biomarkers were selected based on their biological plausibility, non-invasive sampling potential, and the cumulative body of clinical evidence available in early-life populations.

Despite numerous studies published in the literature that have examined these biomarkers individually, there is a lack of a comprehensive consolidation and evaluation on the predictive validity in relation to food allergic disease development. Furthermore, environmental risk factors such as vitamin D status, air pollution, and the composition of the gut microbiome have been implicated in modulating food allergy risk, adding further complexity to the prediction of disease onset [29,30]. The interaction between these biomarkers and their combined predictive value remains insufficiently investigated. A systematic review of the available data is an essential step to providing practical clinical recommendations and outlining knowledge gaps for future research. Accordingly, this review has three specific aims: to synthesize current evidence on each biomarker individually, to compare their relative utility across IgE-mediated and non-IgE-mediated food allergy phenotypes, and to critically appraise the analytical validity of the assays employed, with particular attention to commercially available zonulin ELISA kits whose specificity has recently been questioned. It is hypothesized that these four biomarkers represent distinct but mechanistically linked stages of allergic sensitization, and that only an integrated, multi-marker approach, rather than reliance on any single marker, can meaningfully advance early risk stratification for food allergy.

## 2. Materials and Methods

### 2.1. Study Design and Objective

A systematic review was performed to identify early-life biomarkers that are associated with risk and onset of food allergy in neonates, infants and young children. The review was based on four biomarker domains related to different biological pathways: cord blood IgE (CS-IgE), fecal calprotectin (FC), fecal zonulin (FZ) and secretory IgA (SIgA).

This study was performed according to the Preferred Reporting Items for Systematic Reviews and Meta-Analyses (PRISMA 2020) statement [31]. The review was not prospectively registered in the PROSPERO database, as it was initiated as an academic thesis project. Although the review was not prospectively registered, a prespecified protocol guided all stages of the review. The protocol was subsequently registered in the Open Science Framework (OSF; registration ID: Rt2sx) to enhance transparency and facilitate public access to the review methodology.

### 2.2. Search Strategy

A comprehensive literature search was conducted in the PubMed/MEDLINE and Scopus databases to identify relevant studies published between 1 January 2009 and 20 December 2025. The search strategy combined Medical Subject Headings (MeSH) terms and free-text keywords related to early-life biomarkers and food allergy outcomes.

Separate search strategies were developed for each biomarker category, incorporating terms related to neonatal or infant populations, immunological markers, and food allergy or sensitization outcomes.

The full search strategies are detailed below.

#### 2.2.1. Cord Blood IgE Search Strategy

PubMed: ("Fetal Blood"[Mesh] OR "umbilical cord"[Title/Abstract] OR "cord blood"[Title/Abstract]) AND ("Immunoglobulin E"[Mesh] OR "IgE"[Title/Abstract] OR "total IgE"[Title/Abstract]) AND ("Food Hypersensitivity"[Mesh] OR "food allergy"[Title/Abstract] OR "food sensitization"[Title/Abstract] OR "cow’s milk allergy"[Title/Abstract] OR "egg allergy"[Title/Abstract]) AND ("predict*"[Title/Abstract] OR "prognostic"[Title/Abstract] OR "cohort"[Title/Abstract] OR "longitudinal"[Title/Abstract] OR "risk factor"[Title/Abstract] OR "association"[Title/Abstract]) NOT ("stem cell"[Title/Abstract] OR "transplant*"[Title/Abstract]).

Scopus: ("umbilical cord" OR "cord blood" OR "fetal blood") AND ("IgE" OR "immunoglobulin E" OR "total IgE" OR "specific IgE") AND ("food allergy" OR "food hypersensitivity" OR "food sensitization") AND (predict* OR prognos* OR cohort OR longitudinal OR "risk factor") AND NOT (mice OR rat OR animal OR murine).

#### 2.2.2. Zonulin and Intestinal Permeability Search Strategy

PubMed: ("zonulin"[Title/Abstract] OR "intestinal permeability"[Title/Abstract] OR "gut permeability"[Title/Abstract]) AND ("cord blood"[Title/Abstract] OR "meconium"[Title/Abstract] OR "neonate"[Title/Abstract] OR "newborn"[Title/Abstract] OR "infant"[Title/Abstract]) AND ("food allergy"[Title/Abstract] OR "sensitization"[Title/Abstract] OR "atopy"[Title/Abstract] OR "allergic"[Title/Abstract]).

Scopus: ("zonulin" OR "intestinal permeability") AND ("cord blood" OR "meconium" OR "infant") AND ("food allergy" OR "sensitization").

#### 2.2.3. Calprotectin Search Strategy

PubMed: ("calprotectin"[Title/Abstract] OR "fecal calprotectin"[Title/Abstract]) AND ("infant"[Title/Abstract] OR "newborn"[Title/Abstract]) AND ("cow’s milk allergy"[Title/Abstract] OR "food allergy"[Title/Abstract] OR "allergic colitis"[Title/Abstract]).

Scopus: ("calprotectin" OR "fecal calprotectin" OR "faecal calprotectin") AND ("infant" OR "newborn" OR "neonate" OR "meconium") AND ("food allergy" OR "cow’s milk allergy" OR "allergic colitis").

#### 2.2.4. Secretory IgA Search Strategy

PubMed: ("secretory IgA"[Title/Abstract] OR "sIgA"[Title/Abstract] OR "fecal IgA"[Title/Abstract] OR "mucosal IgA"[Title/Abstract]) AND ("infant"[Title/Abstract] OR "newborn"[Title/Abstract] OR "neonate"[Title/Abstract]) AND ("food allergy"[Title/Abstract] OR "cow’s milk allergy"[Title/Abstract] OR "atopy"[Title/Abstract] OR "sensitization"[Title/Abstract]).

Scopus: ("secretory IgA" OR "sIgA" OR "fecal IgA" OR "faecal IgA" OR "mucosal IgA" OR "salivary IgA") AND ("infant" OR "newborn" OR "neonate") AND ("food allergy" OR "cow’s milk allergy" OR "atopic dermatitis" OR "eczema" OR "sensitization") AND NOT ("mice" OR "mouse" OR "rat" OR "animal").

PubMed/MEDLINE and Scopus were selected as the primary databases for this review because together they provide broad and complementary coverage of the biomedical literature relevant to pediatric allergy and immunology. PubMed/MEDLINE offers comprehensive indexing of clinical and immunological studies, while Scopus extends coverage to additional peer-reviewed journals not indexed in MEDLINE. Although Web of Science (WoS) is another major citation database, previous comparative analyses indicate that the combination of PubMed and Scopus captures the majority of relevant records in biomedical fields, with minimal additional unique yield from WoS. We acknowledge the omission of Web of Science and other databases, such as Embase and CENTRAL, as a potential limitation. Future updates of this review would benefit from their inclusion to further reduce the risk of missing eligible studies.

### 2.3. Study Selection and Screening Process

The literature search only included observational study designs (prospective cohorts, case–control and longitudinal studies) published in English. To ensure methodological rigor and minimize selection bias, the screening process was performed in duplicate by two independent reviewers. After independently assessing the titles and abstracts of all identified records, both reviewers conducted full-text evaluations of articles that may have met eligibility criteria. Disagreements were resolved by consensus, with involvement of a third reviewer when necessary. The same two reviewers then extracted the data using a standardized form that recorded the study year, author, design, target population, biomarkers assessed, reported values, diagnostic thresholds, and effect estimates; disagreements were settled by consensus. No automation techniques were employed, and no communication with research investigators was performed in order to collect or validate data.

**Figure 1:**
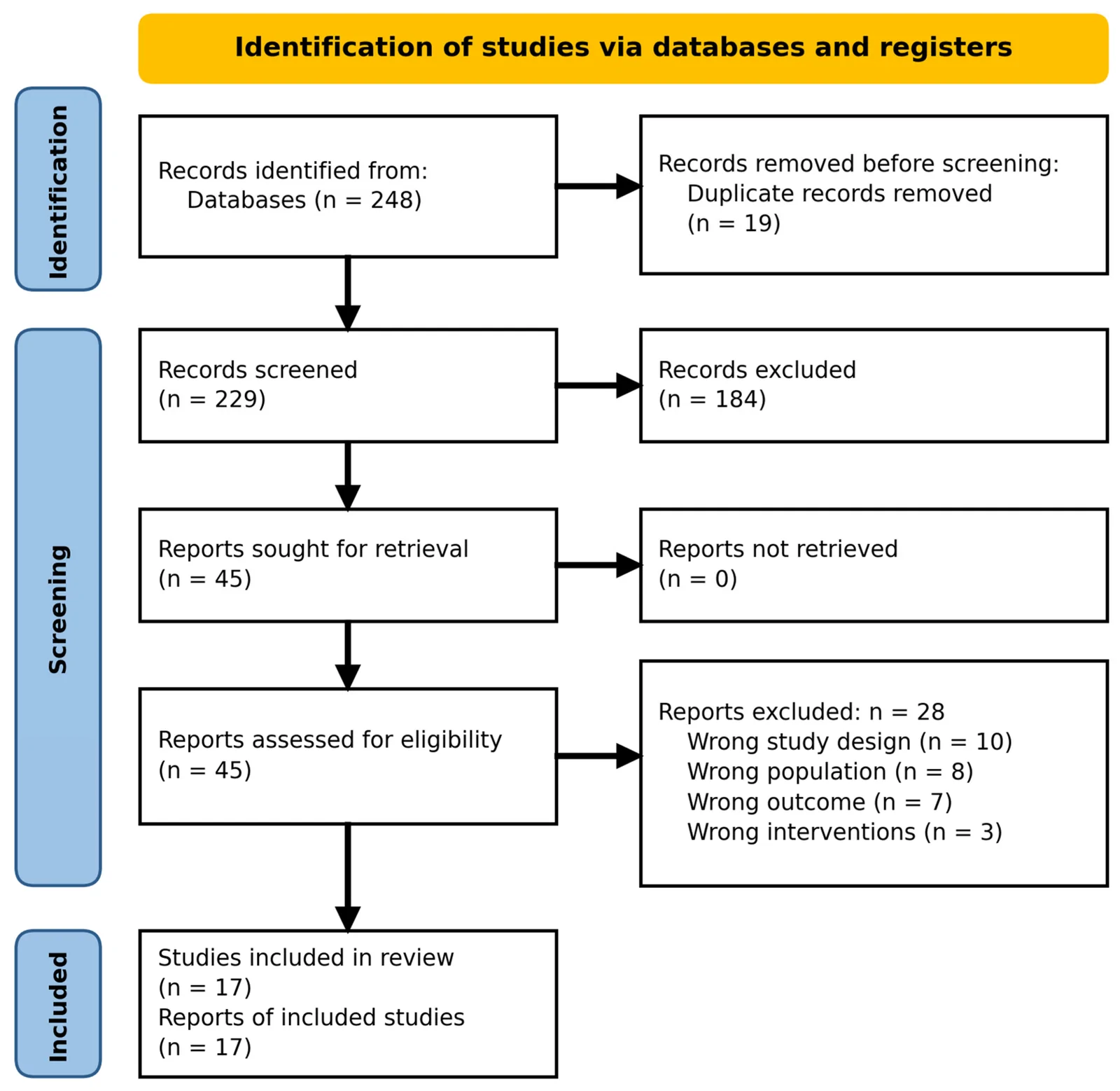
PRISMA Flow Diagram.

The first search identified 248 records. After removing 19 duplicates, 229 articles were left for screening. After title and abstract screening, 45 studies were eligible for full-text review. A total of 17 studies met the study inclusion criteria and were selected in the final qualitative synthesis (Figure 1).

### 2.4. Quality Assessment

The included studies were assessed for methodological quality by Newcastle–Ottawa scale (NOS) [32]. Due to the lack of randomized controlled trials in the review and the substantial methodological heterogeneity that prevented a formal evidence synthesis suitable for GRADE grading, a formal certainty of evidence evaluation utilizing the GRADE system was not carried out. While a close look at the NOS ratings (which range 6 to 9) would indicate an overall high-quality body of data, some these methodological issues are still frequent in the literature:

Selection bias: Many studies used high-risk clinical cohorts, for instance, newborns with severe eczema or a family history of atopy. This restricts the use of biomarker predictive values, especially CS-IgE, to the general unselected pediatric population.

Detection bias: A number of case–control studies reported that clinical outcomes were not evaluated in a transparent manner, which can influence subjective grading of symptoms.

Confounding factors: Average quality studies (NOS scores 5–6) did not always rigorously account for confounding environmental and nutritional factors including exact duration of exclusive breastfeeding and maternal elimination diets.

In summary, the biases indicate that even with established pathophysiological roles for biomarkers such as fecal calprotectin, studies investigating their stand-alone diagnostic properties (sensitivity/specificity) may have slightly overestimated them.

The methodological quality of the included studies is summarized in Table 1.

### 2.5. Eligibility Criteria

#### 2.5.1. Studies Were Included If They Met the Following Criteria

- Human studies with prenatal, newborn, infant or early childhood populations.
- Measurement of at least one biomarker: Cord blood IgE, Fecal calprotectin, Zonulin and intestinal permeability markers, Secretory IgA.
- Results of food allergy, food sensitization, cow’s milk protein allergy, or allergic gastrointestinal illness.
- Observational study designs include cohort, case–control and longitudinal studies.
- Peer-reviewed journal articles in English.

#### 2.5.2. Studies Were Excluded If They Met Any of the Following Criteria

- Use of animal models;
- Reviews, editorials, commentaries, or mechanistic articles without clinical outcomes;
- Focus on therapeutic interventions (e.g., probiotics or treatment studies);
- Inclusion of adult populations;
- Not having relevant data on biomarkers of interest or irrelevant allergy outcomes;
- Publication in languages other than English.

## 3. Results

### 3.1. The Implication of Cord Blood IgE on Food Allergy Development

Cord blood IgE had variable but generally limited predictive value for early-onset food allergies across the included cohort studies.

Importantly, higher CS-IgE levels were associated with an increased long-term risk of atopic disease from the age of five onwards: A threshold ≥ 0.5 kU/L was associated with up to a three-fold increased risk for developing atopic dermatitis (33% vs. 15%) and about four-fold greater odds for symptoms of FA (odds ratio [OR] 4.3; 95% CI 1.2–14.8) [5].

Early Infancy Utility: CS-IgE levels among most infants (>81%) were <0.35 IU/mL regardless of clinical outcome during the first 6 months of life and mean CS-IgE levels did not significantly identify healthy vs. FA newborns (*p* = 0.962) [35].

Antigen-Specific IgE at Birth: Studies of specific IgE (e.g., peanut) have consistently demonstrated that cord blood is lacking detectable allergen-specific antibodies (<0.35 kU/L), including in infants who subsequently developed severe FA. This finding is in agreement with the theory on postnatal transcutaneous sensitization [41].

Phenotypic Association: Complex various phenotypes, particularly the “Food Allergy associated with Atopic Dermatitis” cohort (*p* < 0.01), were strictly correlated with the highest aggregate CS-IgE values [33].

Clinically significant CS-IgE thresholds differed between approximately 0.35 and 0.9 kU/L across studies, with reported sensitivities of about 40–55% and specificities ranging around 65–75%. In summary, cord blood IgE exhibited moderate specificity and low sensitivity supporting the concept of a systemic atopic predisposition marker but questioning its role as an independent food allergy predictor during early childhood. The design, population and main findings of the individual cord blood IgE studies are summarized in Table 2.

**Table 2:** Cord blood IgE studies.

Study Type	Target Population	Main Methodology	Significant Results	Author, Year
**Observational**	Children with peanut allergy (ALSPAC cohort)	Skin prick test + serum peanut-specific IgE (fluoroenzyme immunoassay)	Peanut-specific IgE was always absent at birth (<0.35 kU/L), suggesting sensitization occurs postnatally.	Lack et al., 2003 [41]
**Longitudinal cohort (0–20 years)**	Neonates in Finland	Cord serum total IgE (immunoassay); threshold-based risk analysis	CS-IgE ≥ 0.5 kU/L associated with a 4-fold increased risk of FA symptoms at age 5 (OR 4.3; 95% CI 1.2–14.8).	Pesonen et al., 2009 [5]
**Birth cohort**	741 mother–child pairs (China)	Cord blood total IgE + 25(OH)D3 quantification; group comparison	No significant difference in mean CS-IgE between healthy and allergic infants in the first 6 months (*p* = 0.962).	Wang et al., 2022 [35]
**Cohort (COCOA)**	1699 children (Korea)	Cord blood total IgE; phenotype-based cluster analysis	Highest CS-IgE levels found in the “Food Allergy associated with Atopic Dermatitis” (FA with AD) phenotype (*p* < 0.01).	Lee et al., 2022 [33]

### 3.2. Intestinal Inflammatory and Barrier Biomarkers: Fecal Calprotectin and Zonulin

Fecal calprotectin demonstrated the most consistent and clinically significant association with gastrointestinal food allergies. Across included studies, FC values in active food allergy or milk protein-induced allergic proctocolitis (MPIAP) ranged from approximately 200 to 410 µg/g (with severe cases scoring CoMiSS ≥ 12 showing markedly elevated median values of approximately 2934 µg/g). Healthy control values ranged from approximately 30 to 150 µg/g. Receiver operating characteristic analyses across studies reported sensitivities of 78–98.3% and specificities of 84–93.3%, with optimal diagnostic cut-offs ranging from approximately 193.75 to 486 µg/g depending on assay and population. FC levels showed statistically significant and progressive decline following allergen elimination diets (e.g., from approximately 382 µg/g to 208 µg/g; *p* < 0.05), supporting its role as a disease activity monitoring tool.

Fecal zonulin levels were significantly elevated in infants with MPIAP at presentation (median ~103.6 ng/mL) compared to controls (median ~54.1 ng/mL). A threshold of >66.28 ng/mL demonstrated 100% sensitivity in one study; however, this finding must be interpreted with considerable caution. Commercially available ELISA assays used in the included studies do not specifically detect pre-haptoglobin 2 (the protein originally identified as zonulin) and instead cross-react with structurally related proteins, including properdin and complement factors [20,21]. Critically, none of the 17 included studies employed mass spectrometry-based assays or otherwise validated the analyte specificity of their zonulin measurements. As a consequence, the elevated FZ levels and associated diagnostic accuracy values reported in the zonulin-related studies should be interpreted as reflecting a broader inflammatory or complement-related signal rather than specific intestinal tight junction regulation. This represents a fundamental limitation of the available zonulin evidence base. FZ levels showed dynamic responsiveness following cow’s milk elimination, but demonstrated limited responsiveness during acute FPIES reactions compared to FC. The characteristics and main outcomes of the individual fecal calprotectin and zonulin studies are presented in Table 3.

**Table 3:** Fecal calprotectin and zonulin studies.

Study Type	Target Population	Main Methodology	Significant Results	Author, Year
**Prospective**	57 infants with GI symptoms (Finland)	Fecal calprotectin (ELISA) + cow’s milk-specific Ig; food challenge	Challenge-positive infants had significantly higher FC than challenge-negative infants (55 vs. 29 µg/g; *p* = 0.0039).	Merras-Salmio et al., 2014 [19]
**Prospective case–control**	29 exclusively breastfed infants with allergic colitis	Fecal calprotectin (ELISA); maternal elimination diet monitoring	FC declined following maternal hypoallergenic diet, but showed poor predictive value for symptom improvement (*p* = 0.741).	Ataee et al., 2018 [36]
**Prospective case–control**	90 infants with milk protein allergy (China)	Fecal calprotectin (ELISA); pre/post elimination comparison	Baseline FC significantly higher than controls (410 vs. 141 µg/g; *p* < 0.001) and declined after dietary elimination.	Qiu et al., 2021 [20]
**Case–control**	27 infants with FPIAP (Poland)	Fecal calprotectin + EDN + TNF-α (ELISA); ROC analysis	FC AUC 0.803 (threshold > 486 µg/g); EDN/fCal ratio improved diagnostic accuracy (AUC 0.878).	Rycyk et al., 2023 [42]
**Prospective**	120 children with CMPA (Egypt)	Fecal calprotectin (ELISA) + CoMiSS scoring; correlation analysis	FC significantly correlated with symptom severity (CoMiSS) (R = 0.931); threshold 1700 µg/g had 95.8% accuracy.	Zain-Alabedeen et al., 2023 [37]
**Observational case–control**	34 infants with CMA (Spain)	Fecal calprotectin (ELISA) + 16S microbiota sequencing	FC significantly differentiated allergic from control groups and correlated with microbiota functionality.	Zubeldia-Varela et al., 2023 [38]
**Case–control/Longitudinal**	86 infants with MPIAP (Poland)	Fecal zonulin + calprotectin (ELISA); ROC threshold analysis	FC threshold > 193.75 (Sens 92%) and FZ threshold > 66.28 ng/mL (Sens 100%) distinguished patients from controls.	Czaja-Bulsa et al., 2024 [39]
**Observational**	40 patients (Japan)	Sequential fecal calprotectin (ELISA) post-exposure	Sequential FC ratio to baseline post-exposure showed 90.9% sensitivity on day 1 (threshold 1.532).	Nagata et al., 2024 [43]
**Prospective**	76 infants with OFC-confirmed CMA (Italy)	Fecal calprotectin (ELISA); OFC-confirmed diagnosis	FC significantly decreased from median 30 to 16 mg/kg after 4-week elimination diet (*p* = 0.0014).	Anania et al., 2025 [34]
**Longitudinal comparative**	38 children with FPIES (France)	Fecal calprotectin + zonulin (ELISA); longitudinal comparison	FC useful primarily as a dynamic post-exposure marker; zonulin did not reflect acute reactions.	Lemoine et al., 2025 [40]

### 3.3. Secretory IgA and Mucosal Immune Maturation in Food Allergy

Food allergies and secretory IgA were context-dependent, more reflective of mucosal immune maturation, than direct disease activity.

Aligned mucosal immune maturation was associated with clinical disease. In the first 2 years of life, median salivary SIgA levels in sensitized newborns with allergy symptoms were significantly lower (141 mg/L) than those from asymptomatic sensitized infants (176 mg/L) (*p* < 0.05) [26].

Maternal specific SIgA transfer is tightly associated with protective effects. In this study, lower concentrations of breast milk β-lactoglobulin (BLG)-specific and casein-specific IgA were associated with the infant developing CMPA due to early maternal exclusion diets [27].

Not every infant is protected by high endogenous fecal SIgA. In combination with an aberrant early-life gut microbiota trajectory (early *Bacteroides* depletion: C1–C2 trajectory) and non-exclusive breastfeeding, high SIgA levels synergistically increased the risk of non-atopic wheeze by 4.1-times (95% CI 1.15–14.59), markers suggestive of dysregulated mucosal immune activation [44]. Although this observation relates to a respiratory outcome, a similar pattern of dysregulated mucosal immune activation is mechanistically relevant to food allergy. Elevated SIgA, which arises as a compensatory response to early dysbiosis rather than indicating protective immune exclusion, may mirror a failure of oral tolerance and thus contribute to increased susceptibility to food allergen sensitization.

In general, secretory IgA does not function as a reliable diagnostic biomarker but rather reflects mucosal immune maturation and tolerance development. Findings indicated a context-dependent role mediated by the microbiota and environmental factors. The individual secretory IgA studies are summarized in Table 4.

**Table 4:** Secretory IgA studies.

Study Type	Target Population	Main Methodology	Significant Results	Author, Year
**Prospective comparative**	201 children (Sweden & Estonia)	Salivary SIgA (ELISA); longitudinal maturation analysis	Sensitized children with symptoms had significantly lower SIgA at 3, 6, and 12 months than sensitized asymptomatic children (*p* < 0.05).	Fagerås Böttcher et al., 2011 [26]
**Prospective cohort**	145 mother–child dyads (USA)	Breast milk casein- and BLG-specific IgA (ELISA)	Maternal CM avoidance led to lower casein- and BLG-specific IgA in breast milk (*p* < 0.05), associated with infant CMA.	Järvinen et al., 2014 [28]
**Prospective cohort**	1203 children (CHILD cohort, Canada)	Fecal SIgA (ELISA) + 16S microbiota trajectory analysis	Low *Bacteroides* trajectory (C1–C2) plus high endogenous SIgA (Q4) in non-breastfed infants increased wheeze risk 4.1-fold.	Moore et al., 2025 [27]

A quantitative synthesis of biomarker performance across studies is presented in Table 5.

## 4. Discussion

In this systematic review, biomarker levels at early life (cord blood IgE, fecal markers calprotectin and zonulin, secretory IgA) were assessed for their predictive and diagnostic significance specifically with regard to the onset and clinical activity of food allergy in neonates and young children. These findings demonstrate that, overall, these indicators represent distinct but interconnected pathophysiological pathways involved in allergic disease development. Intestinal biomarkers such as FC and zonulin demonstrate the underlying mucosal inflammation and barrier dysfunction, while SIgA provides insight to mucosal immune maturation and tolerance development. CS-IgE predominantly represents systemic atopic predisposition [3,9,11,23,24]. A recent systematic review of biomarkers in IgE-mediated food allergies confirmed total IgE, specific IgE, and IgG4 as the most commonly reported prognostic markers, while underscoring the limited availability of accessible, standardized biomarkers for routine clinical use [45].

### 4.1. Systemic Atopic Susceptibility Marker (CS-IgE)

The predictive validity of cord blood IgE for future allergy diseases has been debated for years. The results reviewed in this article imply that CS-IgE reflects a broader atopic disposition rather than serving as a reliable independent risk factor for early-onset food allergy. The COCOA cohort data shows a positive association of elevated cord blood IgE with the phenotype “food allergy associated with atopic dermatitis”. Moreover, these findings suggest that CS-IgE identifies children with a more complex systemic atopic trajectory rather than predicts isolated food allergy. Likewise, in a Finnish longitudinal cohort study by Pesonen et al., cord blood IgE levels ≥0.5 kU/L were associated with a ~4 times increased risk of food allergy symptoms during early childhood [5,13,33].

These results are in accordance with previous epidemiological facts indicated by Halken [13] who reported that the best predictive discrimination for the development of allergic disorders later in life is achieved with combination of atopic heredity and elevated cord blood IgE. In this context, CS-IgE is perhaps more a marker of an “atopic constitution”, not constituting clear predictive value for future food allergies, and reflecting atopy-associated immune dysregulation [5,13,17].

In contrast, several biological and methodological factors limit the predictive power of CS-IgE. First, there appears to be limited sensitivity for cord blood IgE as a screening test. Large cohort studies have found sensitivities close to 50%, with a substantial proportion of children who develop allergy disease having normal IgE levels at birth. This limitation is likely due to both methodological constraints and biological variability [16,17,35].

One possible explanation is the particular characteristics of neonatal IgE responses. Consistent with findings by Kawamoto et al. [46], standard immunoassays may fail to detect low-affinity IgE antibodies that are more common in early childhood (e.g., ImmunoCAP). As demonstrated very early on in the landmark study by Lack et al. [41], peanut-specific IgE was undetectable at birth and all participants eventually developed peanut allergy [3], there likely are limitations to detection that may account for the absence of allergen-specific IgE seen in cord blood samples from children later developing clinically relevant food allergies [41,46].

Another important limitation is the discrepancy between immunological sensitization and clinical illness. Gray et al. [47] described the “sensitized-tolerant” phenotype or, as they are sometimes called, “deserters of the atopic march”, who have demonstrable allergen-specific IgE but tolerate those foods without adverse symptomatology. This suggests the potential for overdiagnosis if IgE-based indicators are assessed on their own and further supports that IgE positivity in isolation is not sufficient to discriminate between clinically relevant allergy and asymptomatic sensitisation [12,47].

Environmental exposures may make the assessment of CS-IgE levels far more challenging. For instance, Okada et al. [48] have reported that fetal exposure to perfluorinated compounds such as PFOS and PFOA is associated with reduced IgE production in cord blood. These ambient influences may further limit the predictive value of CS-IgE as a biomarker and cause false-negative screening results in the exposed populations [48].

Taken together, these results indicate that CS-IgE should not be used as a stand-alone test or general screening tool for food allergy. Instead, it may serve as one piece of the puzzle when considered alongside other risk factors, such as early eczema, a family history of atopy, and additional biomarkers that signal gut immune activation [13,33,47]. Prenatal factors, including maternal atopy and environmental exposures during pregnancy, have been shown to influence cord blood IgE levels and neonatal Treg function, adding further complexity to the interpretation of CS-IgE as a predictive marker [49,50].

### 4.2. Intestinal Inflammation and Epithelial Barrier Dysfunction in Food Allergy

Intestinal biomarkers showed a stronger association to gastrointestinal food allergy types (with non-IgE-mediated ones, e.g., milk protein-induced allergic proctocolitis (MPIAP), food protein-induced enterocolitis syndrome (FPIES) and non-IgE-mediated cow’s milk protein allergy (CMPA)) than CS-IgE did in predicting FA [19,20,37,51].

Levels of fecal calprotectin were significantly elevated in allergic gastrointestinal disease infants compared with healthy controls or symptomatic non-allergic children in the studies evaluated. Additionally, FC levels were consistently lower after withdrawing the triggering allergen from the diet, supporting its use as a marker of intestinal inflammation. As shown previously the sensitivities and specificities of FC exceeding 85–90% across several cohorts supports its potential clinical utility as a non-invasive biomarker of such patients in clinical practice [20,34]. Further evidence from a prospective pediatric study confirmed that a strictly followed elimination diet leads to significant reductions in FC levels alongside clinical improvement [52]. Moreover, infants with non-IgE-mediated food allergies exhibit elevated FC levels in parallel with increased Th2 cytokines (IL-4, IL-13), reinforcing the link between intestinal inflammation and systemic immune dysregulation [53].

FC levels correlated with the severity of symptoms. In the cohort of Egyptian newborns with CMPA, fecal calprotectin exhibited a strong positive correlation with the Cow’s Milk-related Symptom Score (CoMiSS), suggesting that FC may be not only an index diagnostic test but also a disease activity and treatment response marker [37].

Intestinal biomarkers can be combined into multimarker techniques to potentially improve their diagnostic efficiency. For instance, in dietary protein-induced allergic proctocolitis in infants, the combination of fecal calprotectin and eosinophil-derived neurotoxin (EDN) significantly increased diagnostic sensitivity. Fecal biomarkers that were positive sequentially after oral meal challenges provided significant sensitivity and specificity for recognizing inflammatory responses in FPIES [43,51].

Zonulin serves as a physiological marker of tight junction dynamics, offering further evidence regarding epithelial barrier disruption. Increased intestinal permeability, as indicated by significantly higher fecal zonulin levels in infants with MPIAP at presentation, is thought to play a key role in the pathogenesis of gastrointestinal food allergies [21,22,39].

Emerging evidence suggests that commercially available ELISA assays may lack specificity for pre-haptoglobin 2 (zonulin), potentially detecting structurally related proteins such as complement factors and properdin. Therefore, elevated fecal zonulin levels should be interpreted with caution, as they may reflect broader inflammatory or complement-related processes rather than specific tight junction regulation. These methodological limitations highlight the need for further validation using more specific analytical techniques, such as mass spectrometry-based assays. Despite this, current evidence supports a potential role of barrier dysfunction in food allergy pathogenesis, although the precise contribution of zonulin remains to be fully elucidated [54,55]. Consistent with this, elevated serum zonulin levels have been reported in children with food allergy, particularly in those with gastrointestinal symptoms and non-IgE-mediated forms [56]. Data from the LEAP trial biomarker substudy found no significant differences in serum zonulin levels between infants with peanut allergy and atopic controls, suggesting that systemic permeability markers may not reliably reflect intestinal barrier function in IgE-mediated allergy [57].

Earlier experimental studies have established the relevance of intestinal barrier transport in allergy sensitization. They state that low dietary polyamine content during early development may impair the maturation of the intestinal barrier, induce permeability and facilitate macromolecule translocation across epithelia. This increased antigen exposure might promote immune sensitization to dietary proteins [44].

Current theories on the development of food allergy also emphasized several possible pathways through which allergen sensitization may occur. The “dual allergen exposure hypothesis” postulates that allergic sensitization can occur via cutaneous exposure through a disrupted skin barrier, particularly in individuals like infants with eczema or filaggrin gene mutations. But studies have shown that the leaky immature intestinal barrier in early life may be an additional route to sensitization. Increased intestinal permeability in infancy may contribute to the loss of oral tolerance [12,41].

### 4.3. Mucosal Immune Maturation and the Regulatory Role of Secretory IgA

Secretory IgA plays a crucial role in the maintenance of oral tolerance and mucosal immune homeostasis. In contrast to inflammatory biomarkers such as calprotectin, SIgA reflects the immunological balance between intestinal microbiota and dietary antigens with host immunity [23,24].

Prospective cohort studies included in this review show a possible correlation between delayed maturation of SIgA production during infancy and allergy symptoms. In the Swedish–Estonian cohort, salivary SIgA levels were significantly lower in sensitized children who developed allergic symptoms during their first year of life compared to those who remained asymptomatic. These findings support the notion that increased SIgA synthesis could protect against excessive penetration of the antigen across the mucosal barrier and increase immunotolerance [26].

SIgA and allergy disease, on the other hand, appear to have a complex and context-dependent relationship. In the Canadian CHILD cohort, interactions between SIgA levels and gut microbiome trajectories as well as newborn feeding patterns modified the risk of respiratory complaints later in life, suggesting that SIgA could, under certain circumstances, perhaps reflect a continuous mucosal immune activation [27].

Another layer of complexity is added by maternal IgA secreted through breast milk. Studies have linked low levels of antigen-specific IgA in breast milk to elevated risk of cow’s milk protein allergy in infants. Experimental models were able to show that breast milk with high levels of IgA can prevent antigens from passing through the intestinal epithelium, further supporting the idea that maternal SIgA is important in maintaining mucosal tolerance early in life [28]. The immunoglobulin profile of human milk varies significantly throughout lactation, with SIgA concentrations remaining elevated even during prolonged breastfeeding, highlighting the continuous passive immune protection provided to the infant [58]. Nevertheless, the role of food-specific IgA remains complex peanut-specific gut IgA does not correlate with protection from peanut allergy, suggesting that IgA functionality is highly context-dependent and influenced by microbiota interactions [59].

### 4.4. Pathophysiological Model of Early-Life Food Allergy

Taken together, these findings indicate that these biomarkers are part of a multi-stage timeline of allergic sensitization and clinical presentation rather than appearing in isolation. Based on the synthesized evidence, the following conceptual framework can be proposed.

Phase 1: Systemic Atopic Predisposition. The major biomarker is Cord Blood IgE (CS-IgE).

Mechanism: CS-IgE reflects early immune conditioning and an underlying atopic constitution. It identifies infants on a systemic atopic trajectory but has low sensitivity for isolated FA [5,13,16,33].

Phase 2: Epithelial Barrier Dysfunction. The primary biomarker is Fecal Zonulin (FZ).

Mechanism: Alteration of gut barrier. Increased intestinal permeability (leaky gut) induced by integrity disappearance of tight-junctions and confirmed with increase in FZ levels.

Consequence: This disruption facilitates increased translocation of dietary antigens across the intestinal epithelium [21,22,39].

Phase 3: Local Mucosal Inflammatory Response. The leading biomarker is fecal calprotectin (FC).

Mechanism: Local inflammatory response to the entrance of allergens, due to neutrophil infiltration and consequent release of FC into the gut lumen.

Clinical Value: FC is strongly correlated with symptom severity and is also the most accurate measure of mucosal inflammation and active disease [18,20,37,39].

Phase 4: Failure of Immune Regulation. The main biomarker is secretory IgA (SIgA).

Mechanism: SIgA is required for “immune exclusion,” meaning blocking antigens before they cross the barrier. Consequently, sensitization progresses to overt clinical allergy because oral tolerance is not induced for either delayed maturation of endogenous SIgA or insufficient maternal SIgA gain [23,24,26,28].

This multi-stage model aligns with the “dual allergen exposure hypothesis” which posits that oral exposure to food antigens promotes tolerance, while cutaneous exposure through a disrupted skin barrier drives sensitization [60]. This paradigm has been instrumental in reshaping food allergy prevention guidelines worldwide [61].

### 4.5. Clinical Implications for Pediatric Practice

The evidence synthesized in this review highlight that early-life biomarkers in food allergies may hold potential clinical relevance, despite their limited current application in standard pediatric practice.

Among the measured biomarkers, fecal calprotectin appears to provide the most encouraging potential for therapeutic application, particularly in non-IgE-mediated gastrointestinal food allergies including cow’s milk protein allergy, food protein-induced allergic proctocolitis and FPIES. Its gradual rise in active disease and decline post elimination diets suggest a potential role in monitoring as well as diagnosis. But as a stand-alone clinical tool, its use now is limited by variability in the assays used and the lack of established cut-off values. Diagnostic thresholds of fecal calprotectin in the future should be strictly age stratified (e.g., 0–3 months vs. 6–12 months) since healthy neonates have much higher physiological gut immaturity and levels compared to older infants [20,34].

Although cord blood IgE serves to assess a general atopic tendency, it lacks sufficient sensitivity or specificity for use as a diagnostic or predictive tool of food allergies. It may be limited to the research context or risk stratification models that integrate clinical covariates [5,13].

While zonulin is a reasonably biologically relevant marker of intestinal barrier failure, due to substantial methodological limitations in terms of assay specificity and reproducibility its clinical application is currently precluded. Zonulin should currently be regarded as an experimental biomarker requiring further validation [21,39].

Secretory IgA reflects the creation of oral tolerance and mucosal immune development, but its interpretation must be done in context and is subject to multiple influencing factors such as breastfeeding patterns and microbiota composition. Consequently, its clinical utility remains limited to research settings [23,28].

Recent advances in multi-omics approaches, combining protein biomarkers with microbiome sequencing and biochemical profiling, show early evidence of improving diagnostic accuracy. For example, integrative analysis of gut microbiota functionality and fecal biomarkers has revealed altered metabolic pathways, such as glycerophospholipid metabolism, in cow’s milk allergic infants [38]. Future predictive algorithms should incorporate such multidimensional data [62].

Overall, none of the evaluated biomarkers can currently be recommended for routine application as separate diagnostic or prognostic tools in food allergy in children. Future studies should focus on large, well-designed prospective trials and assay standardization prior to clinical implementation as well as the establishment of integrated multi-biomarker models.

## 5. Limitations and Future Directions

One of the main limitations of this systematic review is the high methodological variability between included papers that not only made a formal quantitative meta-analysis impossible but also restricts generalizability of any findings from qualitative synthesis. Several key factors contribute to this heterogeneity:

Disparity of Biomarker Assays: Commercially available ELISA-based assays lack standardization and may show variable specificity. This heterogeneity in assays, especially for fecal calprotectin, results in a lack of common cut-off values or units between laboratories.

Age-Dependent Physiological Variances: The physiological immaturity of the gut barrier leads to high baseline levels and great variability in intestinal biomarkers (particularly FC and zonulin) during the initial months after birth, which complicates comparisons across different age groups.

Allergy Diagnostic Criteria: Certain studies used open food challenges (OFC), symptom scores (for instance CoMiSS) or clinical history to define objective allergy, which may have introduced diagnostic bias. Not all used the gold-standard double-blind placebo-controlled food challenge (DBPCFC).

Confounding Allergy Phenotypes: Several studies combined data on IgE-mediated and non-IgE-mediated food allergies. Mucosal inflammatory markers, which are predominantly elevated in non-IgE disorders, have less specific predictive ability for prognosis due to the fact they derive from different pathophysiological pathways.

Publication Bias: Publication bias may have had a role in the results as research reporting positive correlations between biomarkers and outcomes of food allergies is more likely to be published.

Limited generalizability: The results may not be as transferrable to the broader pediatric population because most of the included studies were conducted in select subjects, such as newborns with atopic dermatoses or family history of allergies.

Future studies should primarily focus on large, prospective birth cohorts that integrate multiple biomarker domains with clinical and environmental risk factors. Development of such integrative multimarker predictive models will be important for the early risk stratification of food allergies. Importantly, next-generation risk prediction algorithms will require the integration of these classical protein biomarkers (immunological and intestinal), advanced microbiome sequencing techniques (such as 16S rRNA or shotgun metagenomics) and environmental exposure data for optimal diagnostic accuracy. In addition, the establishment of age-specific reference ranges and standardization of biomarker testing will also be required to translate these results into clinical practice.

In accordance with PRISMA 2020 item 16b, a complete list of individually excluded studies with specific reasons could not be retrospectively reconstructed. The primary reasons for exclusion at the full-text review stage were consistently applied and are as follows: use of animal models; inclusion of adult-only populations; focus on therapeutic or interventional outcomes; absence of relevant biomarker data for the outcomes of interest; publication in languages other than English. The exclusion categories were pre-specified in Section 2.5.2 and applied consistently and independently by two reviewers during the full-text screening stage. The most frequent reason for exclusion at the full-text stage was the absence of extractable biomarker data related to food allergy or sensitization outcomes. Other common reasons included the inclusion of adult or mixed-age populations without pediatric-specific data and a focus on therapeutic interventions, such as probiotic or immunotherapy trials, rather than on the diagnostic or predictive value of the biomarker. Studies utilizing animal models and non-English publications were excluded at earlier stages. While PRISMA 2020 item 16b recommends a fully itemized list of excluded reports, such a list could not be retrospectively reconstructed for this review. This is acknowledged as a reporting limitation.

## 6. Conclusions

This systematic review demonstrates that early-life biomarkers, CS-IgE, fecal calprotectin, fecal zonulin, and secretory IgA, reflect distinct but interconnected biological pathways involved in food allergy development, including systemic atopic predisposition, epithelial barrier dysfunction, intestinal inflammation, and mucosal immune regulation. None of the evaluated biomarkers currently demonstrates sufficient standalone diagnostic or predictive accuracy to support routine clinical implementation. Fecal calprotectin shows the most consistent evidence for clinical relevance in non-IgE-mediated gastrointestinal food allergies, while fecal zonulin requires fundamental assay re-validation before its clinical utility can be established. The greatest potential for improving early risk stratification of food allergy lies in integrated, validated multi-biomarker models combined with clinical risk factors. Future large-scale prospective studies employing standardized methodologies are essential to validate this approach and enable clinical translation.

## Figures and Tables

**Table 1:** Newcastle–Ottawa Scale (NOS) scores for included studies.

Study (Author, Year)	Selection (Max 4)	Comparability (Max 2)	Outcome/Exposure (Max 3)	Total Score
Järvinen et al., 2014 [28]	4	2	3	9
Moore et al., 2025 [27]	4	2	3	9
Merras-Salmio et al., 2014 [19]	4	2	3	9
Pesonen et al., 2009 [5]	4	2	3	9
Lee et al., 2022 [33]	4	2	3	9
Qiu et al., 2021 [20]	3	2	3	8
Anania et al., 2025 [34]	3	2	3	8
Wang et al., 2022 [35]	3	2	3	8
Fagerås et al., 2011 [26]	3	1	3	7
Ataee et al., 2018 [36]	3	1	3	7
Zain-Alabedeen et al., 2023 [37]	3	1	3	7
Zubeldia-Varela et al., 2023 [38]	3	1	3	7
Czaja-Bulsa et al., 2024 [39]	3	1	3	7
Lemoine et al., 2025 [40]	3	1	3	7
Lack et al., 2003 [41]	3	1	3	7
Rycyk et al., 2023 [42]	3	1	2	6
Nagata et al., 2024 [43]	3	0	3	6

**Table 5:** Reported Ranges of Biomarker Performance Across Included Studies in Early-Life Food Allergy.

Biomarker	Clinical Phenotype/Target	Reported Values in Allergy (Median Range)	Reported Ranges Across Included Studies	Proposed Cut-Offs	Diagnostic Accuracy (Sens./Spec./AUC)
Fecal Calprotectin (FC)	Non-IgE FA (MPIAP, FPIES, CMPA)	~200–410 µg/g (Severe CoMiSS ≥ 12: ~2934 µg/g)	~30–150 µg/g	>193.75 to >1700 µg/g	Sens.: 78–98.3% Spec.: 84–93.3% AUC: 0.803
FC + EDN Ratio	FPIAP (Mucosal Inflammation)	Elevated	Baseline	>884 ng/mL (EDN)	Sens.: 89% Spec.: 84% AUC: 0.878
Fecal Zonulin (FZ)	MPIAP (Intestinal Permeability)	~103.6 ng/mL	~54.1 ng/mL	>66.28 ng/mL	Sens.: 100% Spec.: Not consistently reported
Cord Blood IgE (CS-IgE)	Systemic Atopy/Complex FA + AD phenotype	High proportion ≥ 0.5 kU/L	Majority < 0.35 kU/L	≥0.5 kU/L	Sens.: ~40–55% Risk: 4-fold increase for FA symptoms (OR 4.3)
Salivary SIgA	Sensitization with clinical symptoms	~141 mg/L	~176 mg/L (Asymptomatic sensitized)	N/A (Prognostic marker)	Correlated with delayed mucosal immune maturation (*p* < 0.05)
Breast Milk-Specific IgA	Maternal immune transfer (CMPA risk)	Undetectable/Low	High	N/A (Prognostic marker)	Low levels strongly predict infant CMPA (*p* < 0.05)

Legend: FA = Food Allergy; MPIAP = Milk Protein-Induced Allergic Proctocolitis; FPIES = Food Protein-Induced Enterocolitis Syndrome; FPIAP = Food Protein-Induced Allergic Proctocolitis; CMPA = Cow’s Milk Protein Allergy; EDN = Eosinophil-Derived Neurotoxin; AD = Atopic Dermatitis; Sens. = Sensitivity; Spec. = Specificity; AUC = Area Under the Receiver Operating Characteristic Curve. N/A = not applicable, i.e., no diagnostic cut-off was reported because the biomarker was evaluated as a prognostic rather than a diagnostic marker. Values represent aggregated medians and ranges derived from the included observational cohorts and case–control studies.

## Data Availability

No new data were created or analyzed in this study. Data sharing is not applicable to this article.

## References

[1] Peters R.L., Mavoa S., Koplin J.J. (2022). An Overview of Environmental Risk Factors for Food Allergy. Int. J. Environ. Res. Public Health.

[2] Nwaru B.I., Hickstein L., Panesar S.S., Muraro A., Werfel T., Cardona V., Dubois A.E.J., Halken S., Hoffmann-Sommergruber K., Poulsen L.K. (2014). The epidemiology of food allergy in Europe: A systematic review and meta-analysis. Allergy.

[3] Sicherer S.H., Sampson H.A. (2014). Food allergy: Epidemiology, pathogenesis, diagnosis, and treatment. J. Allergy Clin. Immunol..

[4] Yu W., Freeland D.M.H., Nadeau K.C. (2016). Food allergy: Immune mechanisms, diagnosis and immunotherapy. Nat. Rev. Immunol..

[5] Pesonen M., Kallio M.J., Siimes M.A., Elg P., Björksten F., Ranki A. (2009). Cord serum immunoglobulin E as a risk factor for allergic symptoms and sensitization in children and young adults. Pediatr. Allergy Immunol..

[6] Ballegaard A.S.R., Bøgh K.L. (2023). Intestinal protein uptake and IgE-mediated food allergy. Food Res. Int..

[7] Ogulur I., Pat Y., Aydin T., Yazici D., Rückert B., Peng Y., Kim J., Radzikowska U., Westermann P., Sokolowska M. (2023). Gut epithelial barrier damage caused by dishwasher detergents and rinse aids. J. Allergy Clin. Immunol..

[8] Iglesia E.G.A., Kwan M., Virkud Y.V., Iweala O.I. (2024). Management of Food Allergies and Food-Related Anaphylaxis. JAMA.

[9] Crespo J.F., Cabanillas B. (2023). Recent advances in cellular and molecular mechanisms of IgE-mediated food allergy. Food Chem..

[10] Vickery B.P., Chin S., Burks A.W. (2011). Pathophysiology of food allergy. Pediatr. Clin. N. Am..

[11] Nowak-Węgrzyn A., Katz Y., Mehr S.S., Koletzko S. (2015). Non-IgE-mediated gastrointestinal food allergy. J. Allergy Clin. Immunol..

[12] Du Toit G., Foong R.-X.M., Lack G. (2016). Prevention of food allergy—Early dietary interventions. Allergol. Int..

[13] Halken S. (2004). Prevention of allergic disease in childhood: Clinical and epidemiological aspects of primary and secondary allergy prevention. Pediatr. Allergy Immunol..

[14] Sampson H.A. (2004). Update on food allergy. J. Allergy Clin. Immunol..

[15] Breiteneder H., Diamant Z., Eiwegger T., Fokkens W.J., Traidl-Hoffmann C., Nadeau K., O’Hehir R.E., O’Mahony L., Pfaar O., Torres M.J. (2019). Future research trends in understanding the mechanisms underlying allergic diseases for improved patient care. Allergy.

[16] Nissen S.P., Kjaer H.F., Høst A., Nielsen J., Halken S. (2015). Can family history and cord blood IgE predict sensitization and allergic diseases up to adulthood?. Pediatr. Allergy Immunol..

[17] Croner S., Kjellman N.-I.M. (1990). Development of atopic disease in relation to family history and cord blood IgE levels. Pediatr. Allergy Immunol..

[18] Beşer Ö.F., Sancak S., Erkan T., Kutlu T., Çokuğraş H., Çokuğraş F.Ç. (2014). Can Fecal Calprotectin Level Be Used as a Marker of Inflammation in the Diagnosis and Follow-Up of Cow’s Milk Protein Allergy?. Allergy Asthma Immunol. Res..

[19] Merras-Salmio L., Kolho K.-L., Pelkonen A.S., Kuitunen M., Mäkelä M.J., Savilahti E. (2014). Markers of gut mucosal inflammation and cow’s milk specific immunoglobulins in non-IgE cow’s milk allergy. Clin. Transl. Allergy.

[20] Qiu L., Wang J., Ren F., Shen L., Li F. (2021). Can fecal calprotectin levels be used to monitor infant milk protein allergies?. Allergy Asthma Clin. Immunol..

[21] Fasano A. (2011). Zonulin and its regulation of intestinal barrier function: The biological door to inflammation, autoimmunity, and cancer. Physiol. Rev..

[22] Fasano A., Not T., Wang W., Uzzau S., Berti I., Tommasini A., Goldblum S.E. (2000). Zonulin, a newly discovered modulator of intestinal permeability, and its expression in coeliac disease. Lancet.

[23] Cerutti A., Chen K., Chorny A. (2011). Immunoglobulin responses at the mucosal interface. Annu. Rev. Immunol..

[24] Brandtzaeg P. (2010). Update on mucosal immunoglobulin A in gastrointestinal disease. Curr. Opin. Gastroenterol..

[25] Kukkonen K., Kuitunen M., Haahtela T., Korpela R., Poussa T., Savilahti E. (2010). High intestinal IgA associates with reduced risk of IgE-associated allergic diseases. Pediatr. Allergy Immunol..

[26] Fagerås M., Tomičić S., Voor T., Björkstén B., Jenmalm M.C. (2011). Slow salivary secretory IgA maturation may relate to low microbial pressure and allergic symptoms in sensitized children. Pediatr. Res..

[27] Moore L.E., Tun H.M., Field C.J., Mandhane P.J., Simons E., Moraes T.J., Subbarao P., Turvey S.E., Hicks M., Hicks A. (2025). Secretory IgA modifies the association between early-life gut microbiota trajectories and childhood nonatopic wheeze. ERJ Open Res..

[28] Järvinen K.M., Westfall J.E., Seppo M.S., James A.K., Tsuang A.J., Feustel P.J., Sampson H.A., Berin C. (2014). Role of maternal elimination diets and human milk IgA in the development of cow’s milk allergy in the infants. Clin. Exp. Allergy.

[29] Zhang Q., Chen M., Li Y., Wang X., Liu H., Zhao J., Yang L., Sun J., Zhou X., Zhang L. (2021). Antibiotic-Induced Gut Microbiota Dysbiosis Damages the Intestinal Barrier, Increasing Food Allergy in Adult Mice. Nutrients.

[30] Grijincu M., Buzan M.R., Zbîrcea L.E., Păunescu V., Panaitescu C. (2024). Prenatal Factors in the Development of Allergic Diseases. Int. J. Mol. Sci..

[31] Page M.J., McKenzie J.E., Bossuyt P.M., Boutron I., Hoffmann T.C., Mulrow C.D., Shamseer L., Tetzlaff J.M., Akl E.A., Brennan S.E. (2021). The PRISMA 2020 statement: An updated guideline for reporting systematic reviews. BMJ.

[32] Wells G.A., Shea B., O’Connell D., Peterson J., Welch V., Losos M., Tugwell P. (2021). The Newcastle–Ottawa Scale (NOS) for Assessing the Quality of Nonrandomised Studies in Meta-Analyses.

[33] Lee S.-Y., Kim S., Kang M.J., Song K.-B., Choi E.J., Jung S., Yoon J.-S., Suh D.I., Shin Y.H., Kim K.W. (2022). Phenotype of Atopic Dermatitis with Food Allergy Predicts Development of Childhood Asthma via Gut Wnt Signaling. Allergy Asthma Immunol. Res..

[34] Anania C., Mondì F., Brindisi G., Spagnoli A., De Canditiis D., Gesmini A., Marchetti L., Fichera A., Piccioni M.G., Zicari A.M. (2025). Fecal Calprotectin Determination in a Cohort of Children with Cow’s Milk Allergy. Nutrients.

[35] Wang N.-R., Liu S.-J., Xiao G.-Y., Zhang H., Huang Y.-J., Wang L., He C.-Y. (2022). Cord blood 25(OH)D3, cord blood total immunoglobulin E levels, and food allergies in infancy: A birth cohort study in Chongqing, China. World Allergy Organ. J..

[36] Ataee P., Zoghali M., Nikkhoo B., Ghaderi E., Mansouri M., Nasiri R., Eftekhari K. (2018). Diagnostic value of fecal calprotectin in response to mother’s diet in breast-fed infants with cow’s milk allergy colitis. Iran. J. Pediatr..

[37] Zain-Alabedeen S., Kamel N., Amin M., Vernon-Roberts A., Day A.S., Khashana A. (2023). Fecal Calprotectin and Cow’s Milk-Related-Symptoms Score in Children with Cow’s Milk Protein Allergy. Pediatr. Gastroenterol. Hepatol. Nutr..

[38] Zubeldia-Varela E., Barker-Tejeda T.C., Mera-Berriatua L., Bazire R., Cabrera-Freitag P., Ubeda C., Barber D., Francino M.P., Rojo D., Ibáñez-Sandín M.D. (2023). Further insights into the gut microbiota of cow’s milk allergic infants: Analysis of microbial functionality and its correlation with three fecal biomarkers. Int. J. Mol. Sci..

[39] Czaja-Bulsa G., Bulsa K., Łokieć M., Drozd A. (2024). Can faecal zonulin and calprotectin levels be used in the diagnosis and follow-up in infants with milk protein-induced allergic proctocolitis?. Nutrients.

[40] Lemoine A., Kapel N., Nicolis I., Tounian P., Bruneau A., Kapandji N., Adel-Patient K., Thomas M. (2025). Identification of Gut Biomarkers of FPIES in a Longitudinal Comparative Pediatric Study. Allergy.

[41] Lack G., Fox D., Northstone K., Golding J. (2003). Avon Longitudinal Study of Parents and Children Study Team. Factors associated with the development of peanut allergy in childhood. N. Engl. J. Med..

[42] Rycyk A., Cudowska B., Lebensztejn D.M. (2020). Eosinophil-derived neurotoxin, tumor necrosis factor alpha, and calprotectin as non-invasive biomarkers of food protein-induced allergic proctocolitis in infants. J. Clin. Med..

[43] Nagata M., Inage E., Yamada H., Kudo T., Toriumi S., Sakaguchi K., Tanaka Y., Jimbo K., Ohtsuka Y., Shimizu T. (2024). Efficacy of sequential fecal-marker examination for evaluating gastrointestinal inflammation in solid food protein-induced enterocolitis syndrome. Allergol. Int..

[44] Dandrifosse G., Peulen O., El Khefif N., Deloyer P., Dandrifosse A.C., Grandfils C. (2000). Are milk polyamines preventive agents against food allergy?. Proc. Nutr. Soc..

[45] Malucelli M., Farias R., Mello R.G., Prando C. (2023). Biomarkers associated with persistence and severity of IgE-mediated food allergies: A systematic review. J. Pediatr..

[46] Kawamoto N., Kamemura N., Kido H., Fukao T. (2017). Detection of ovomucoid-specific low-affinity IgE in infants and its relationship to eczema. Pediatr. Allergy Immunol..

[47] Gray L.E.K., Ponsonby A.-L., Collier F., O’Hely M., Sly P.D., Ranganathan S., Tang M.L.K., Carlin J.B., Saffery R., Vuillermin P.J. (2020). Deserters on the atopic march: Risk factors, immune profile and clinical outcomes of food sensitized–tolerant infants. Allergy.

[48] Okada E., Sasaki S., Saijo Y., Washino N., Miyashita C., Kobayashi S., Konishi K., Ito Y.M., Ito R., Nakata A. (2012). Prenatal exposure to perfluorinated chemicals and relationship with allergies and infectious diseases in infants. Environ. Res..

[49] Chen M., Gu Y., Liu Q., Ji R., Zhang J., Hua L. (2021). Relationship of cord blood IgE with maternal, fetal, and environmental factors in the Chinese population. Allergol. Immunopathol..

[50] Černý V., Novotná O., Petrásková P., Hudcová K., Boráková K., Prokešová L., Kolářová L., Hrdý J. (2021). Lower Functional and Proportional Characteristics of Cord Blood Treg of Male Newborns Compared with Female Newborns. Biomedicines.

[51] Roca M., Donat E., Rodriguez Varela A., Carvajal E., Cano F., Armisen A., Ekoff H., Cañada-Martínez A.J., Rydell N., Ribes-Koninckx C. (2021). Fecal Calprotectin and Eosinophil-Derived Neurotoxin in Children with Non-IgE-Mediated Cow’s Milk Protein Allergy. J. Clin. Med..

[52] Lendvai-Emmert D., Emmert V., Makai A., Fusz K., Prémusz V., Eklics K., Sarlós P., Tóth P., Amrein K., Tóth G. (2022). Fecal calprotectin levels in pediatric cow’s milk protein allergy. Front. Pediatr..

[53] Savino F., Giuliani F., Giraudi S., Galliano I., Montanari P., Daprà V., Bergallo M. (2022). Analysis of Serum Th2 Cytokines in Infants with Non-IgE Mediated Food Allergy Compared to Healthy Infants. Nutrients.

[54] Scheffler L., Crane A., Heyne H., Tönjes A., Schleinitz D., Ihling C.H., Stumvoll M., Freire R., Fiorentino M., Fasano A. (2018). Widely used commercial ELISA does not detect precursor of haptoglobin2, but recognizes properdin as a potential second member of the zonulin family. Front. Endocrinol..

[55] Ajamian M., Steer D., Rosella G., Gibson P.R. (2019). Serum zonulin as a marker of intestinal mucosal barrier function: May not be what it seems. PLoS ONE.

[56] Niewiem M., Grzybowska-Chlebowczyk U. (2022). Assessment of selected intestinal permeability markers in children with food allergy depending on the type and severity of clinical symptoms. Nutrients.

[57] Aktas O.N., Mateja A., Li M., Chatman L., Grieco M.C., Baloh C.H., Huffaker M., Wheatley L.M., du Toit G., Lack G. (2025). Evaluation of intestinal permeability using serum biomarkers in the Learning Early About Peanut Allergy trial. Allergy.

[58] Czosnykowska-Łukacka M., Lis-Kuberka J., Królak-Olejnik B., Orczyk-Pawiłowicz M. (2020). Changes in Human Milk Immunoglobulin Profile During Prolonged Lactation. Front. Pediatr..

[59] Liu E.G., Zhang B., Martin V., Anthonypillai J., Kraft M., Grishin A., Grishina G., Chinthrajah R.S., Sindher T., Manohar M. (2022). Food-specific immunoglobulin A does not correlate with natural tolerance to peanut or egg allergens. Sci. Transl. Med..

[60] Yamamoto-Hanada K., Ohya Y. (2023). Skin and oral intervention for food allergy prevention based on dual allergen exposure hypothesis. Clin. Exp. Pediatr..

[61] Turner P.J., Arasi S., Ballmer-Weber B., Conrado A.B., Deschildre A., Gerdts J., Halken S., Muraro A., Patel N., Van Ree R. (2022). Risk factors for severe reactions in food allergy: Rapid evidence review with meta-analysis. Allergy.

[62] Scheurer S., Junker A.C., He C., Schülke S., Toda M. (2023). The role of IgA in the manifestation and prevention of allergic immune responses. Curr. Allergy Asthma Rep..

